# Sedentary behavior and anxiety-depressive disorders among Tunisian Administrative Workers

**DOI:** 10.1192/j.eurpsy.2026.11446

**Published:** 2026-07-31

**Authors:** I. Kacem, A. Ghenim, A. Chouchane, A. Aloui, C. Sridi, F. Chelly, S. Kooli, O. El Maalel, M. Maoua, N. Mrizak

**Affiliations:** 1 Occupational Medicine and Professional Pathologies Department, Farhat Hached Teaching Hospital; 2 Research Laboratory LR19SP03, University of Sousse, Faculty of Medicine of Sousse; 3 Occupational Medicine and Professional Pathologies Department, Sahloul teaching hospital, Sousse, Tunisia

## Abstract

**Introduction:**

Beyond its metabolic and cardiovascular effects, sedentary behavior has gained increasing attention in relation to mental health. Recent studies suggest that it contributes to the development or worsening of psychological disorders, particularly anxiety and depression.

**Objectives:**

To assess the impact of sedentary behavior on psychological health, specifically in terms of perceived stress as well as anxiety and depressive symptoms, among administrative employees of the Tunisian Electricity and Gas Company (STEG) in the Sousse region.

**Methods:**

This descriptive and analytical cross-sectional epidemiological study was conducted among administrative employees of the Tunisian Electricity and Gaz Company “TEGC” across four districts in Sousse, between October and December 2023. Data collection was performed using a self-administered questionnaire and a clinical examination. Physical activity was assessed using the long version of the International Physical Activity Questionnaire (IPAQ). Work-related stress was evaluated with the Perceived Stress Scale (PSS), while anxiety and depression were measured using the Hospital Anxiety and Depression Scale (HADS).

**Results:**

Among the 131 participants, 64% were male, and the mean age was 43.3 ± 9.8 years. The median total physical activity score (IPAQ) over one week was 2843 MET-min/week. Based on the categorical classification of the IPAQ, 22.1% were classified as inactive, 50.4% had minimal activity, and 27.5% were highly active. Regarding perceived stress, 90.8% experienced moderate to high stress compared to 9.1% with low stress. The mean perceived stress score was 23.4 ± 7.5 (median = 24). Seventy-seven participants were diagnosed with probable anxiety and five with possible anxiety, while 20 participants had probable depression and 18 possible depression. In multivariate analysis, a low categorical physical activity score, reflecting increased sedentary behavior, was significantly associated with the presence of possible or probable stress (aOR = 3.889, 95% CI [2.299–6.579], p < 0.001), indicating that more sedentary individuals were nearly four times more likely to experience possible or probable stress compared to less sedentary participants. The categorical sedentary score (aOR = 1.301, 95% CI [0.858–1.974], p = 0.215) did not show a significant effect on anxiety risk. Overall, higher total physical activity scores appeared as protective factor, with decreasing risk of depressive disorders as mean physical activity increase.

**Conclusions:**

This study highlights the significant relationship between sedentary behavior and anxiety-depressive disorders among administrative employees. These findings underscore the importance of promoting regular physical activity and reducing sedentary time in the workplace as strategies to prevent anxiety-depressive disorders.

**Disclosure of Interest:**

None Declared

